# Type 1 diabetes susceptibility alleles are associated with distinct alterations in the gut microbiota

**DOI:** 10.1186/s40168-018-0417-4

**Published:** 2018-02-17

**Authors:** Jane A. Mullaney, Juliette E. Stephens, Mary-Ellen Costello, Cai Fong, Brooke E. Geeling, Patrick G. Gavin, Casey M. Wright, Timothy D. Spector, Matthew A. Brown, Emma E. Hamilton-Williams

**Affiliations:** 1 Translational Research Institute, The University of Queensland Diamantina Institute, University of Queensland, Brisbane, QLD Australia; 2 AgResearch Limited, Grasslands Research Centre, Palmerston North, New Zealand; 3 Institute of Health and Biomedical Innovation, Queensland University of Technology, Translational Research Institute, Brisbane, QLD Australia; 4 Department of Twin Research and Genetic Epidemiology, King’s College London, London, SE1 7EH UK; 5 Translational Research Institute, The University of Queensland Diamantina Institute, 37 Kent St, Woolloongabba, QLD 4102 Australia

**Keywords:** Gut microbiota, Type 1 diabetes, Genetic susceptibility, Interleukin-2 pathway, Autoimmunity

## Abstract

**Background:**

Dysbiosis of the gut microbiota has been implicated in the pathogenesis of many autoimmune conditions including type 1 diabetes (T1D). It is unknown whether changes in the gut microbiota observed in T1D are due to environmental drivers, genetic risk factors, or both. Here, we have performed an analysis of associations between the gut microbiota and T1D genetic risk using the non-obese diabetic (NOD) mouse model of T1D and the TwinsUK cohort.

**Results:**

Through the analysis of five separate colonies of T1D susceptible NOD mice, we identified similarities in NOD microbiome that were independent of animal facility. Introduction of disease protective alleles at the *Idd3* and *Idd5* loci (*IL2*, *Ctla4*, *Slc11a1*, and *Acadl*) resulted in significant alterations in the NOD microbiome. Disease-protected strains exhibited a restoration of immune regulatory pathways within the gut which could also be reestablished using IL-2 therapy. Increased T1D disease risk from IL-2 pathway loci in the TwinsUK cohort of human subjects resulted in some similar microbiota changes to those observed in the NOD mouse.

**Conclusions:**

These findings demonstrate for the first time that type 1 diabetes-associated genetic variants that restore immune tolerance to islet antigens also result in functional changes in the gut immune system and resultant changes in the microbiota.

**Electronic supplementary material:**

The online version of this article (10.1186/s40168-018-0417-4) contains supplementary material, which is available to authorized users.

## Background

Dysbiosis of the gut microbiota has been described in multiple inflammatory and immune-mediated diseases including rheumatoid arthritis, ulcerative colitis, Crohn’s disease ankylosing spondylitis, and type 1 diabetes (T1D). The microbiota is established in the early part of life, and its composition is shaped by deterministic niche-related factors (host environment), historical factors (pre-exposure to certain microbes), and environmental drivers (e.g., diet) [1]. This complexity suggests that the causes of dysbiosis in diseases such as T1D are likely to be multifactorial. As the incidence of autoimmune diseases including T1D has been steadily increasing over the past 50 years [2], it is speculated that lifestyle changes are causing changes in the gut microbiota leading to alterations in the development of the immune system [3]. However, it is unknown whether genetic susceptibility to T1D also contributes to shaping the microbial communities of high-risk individuals, and whether the enteric dysbiosis seen in T1D is a cause or effect of the disease, and the immune dysregulation associated with its development.

A role for the microbiota in T1D etiology is suggested by studies that demonstrate changes in the microbial communities prior to clinical disease onset. Early studies suggested that high-risk, islet-autoantibody-positive children had altered bacterial diversity when compared with low-risk autoantibody-negative children [4, 5]. A recent longitudinal study of Finnish children at risk of T1D found a drop in bacterial diversity prior to the development of clinical disease [6]. Similarly, a cross-sectional study from a US-based cohort found differences in the bacterial composition between new-onset patients, high- and low-risk first-degree relatives, and healthy, unrelated subjects [7]. These data suggest that reduced diversity and/or a change in metabolic function of the gut microbiota may be associated with disease progression in T1D.

In the inbred non-obese diabetic (NOD) mouse model of T1D, susceptible NOD mice were shown to harbor a distinct gut microbiota to a variety of T1D-protected inbred strains [8]. T1D incidence is higher in specific pathogen-free (SPF) or “clean” housing facilities, and many interventions that alter the microbiota (e.g., diet changes) result in disease protection [9]. Exposure to antibiotic regimens early in development both dramatically shifted the bacterial abundance and led to accelerated disease [10]. These studies indicate that in the steady state, NOD mice harbor a “diabetes-permissive” microbiota, and major perturbations in the microbiota can both reduce and accelerate disease.

In other autoimmune diseases such as Crohn’s disease, specific genetic loci associated with disease susceptibility have been shown to influence the gut environment and lead to shifts in the resident microbial populations [11]. To date, such associations have not been investigated in T1D. Genome-wide association studies have identified 58 genetic associations with T1D [12]. These include many genes involved in both adaptive and innate immune pathways [12]. The human leukocyte antigen (HLA)-DQ and HLA-DRB1 major histocompatibility complex (MHC) class II region genes have the strongest association with disease susceptibility [13]. The interleukin-2 (IL-2) signaling pathway, which is linked to regulatory T cell (Treg) function, is strongly implicated in disease pathogenesis due to both a cluster of associated genes and a suite of mechanistic studies in mice [14]. Treg residents in the gut contribute to controlling tolerance to the commensal microbiota [15]; hence, T1D-associated variants in IL-2 pathway genes which reduce Treg function could potentially lead to changes in the microbiota.

In this study, we have investigated whether the gut microbiota of the NOD strain is genetically determined. We utilized NOD mice from multiple animal facilities and congenic NOD mice that carry protective alleles of T1D susceptibility alleles to investigate whether T1D predisposing host genetics modifies the gut microbiota through alterations in the gut immune environment. The regions we investigated included the *MHC* and the *Idd3* and *Idd5* loci which are linked to the IL-2 pathway and Treg function [16–18]. We also investigate whether therapeutic treatment of NOD mice with IL-2, which improves Treg function and mimics the genetic effect of protective *Idd3* alleles [17], reduces inflammation in the gut and restores the microbiota to that found in disease-protected mice. To determine whether T1D-associated risk alleles are associated with alterations in the gut microbiota in human subjects, we utilized the TwinsUK cohort of individuals with matching data on genetic variation and microbiota composition. Our findings for the first time demonstrate a role for T1D susceptibility alleles in shaping the gut microbiota, independently of the presence of T1D, consistent with the gut microbiota having a causative role in the development of T1D.

## Results

### NOD mice have a common structure to their gut microbiome that is independent of animal facility

**Fig. 1:**
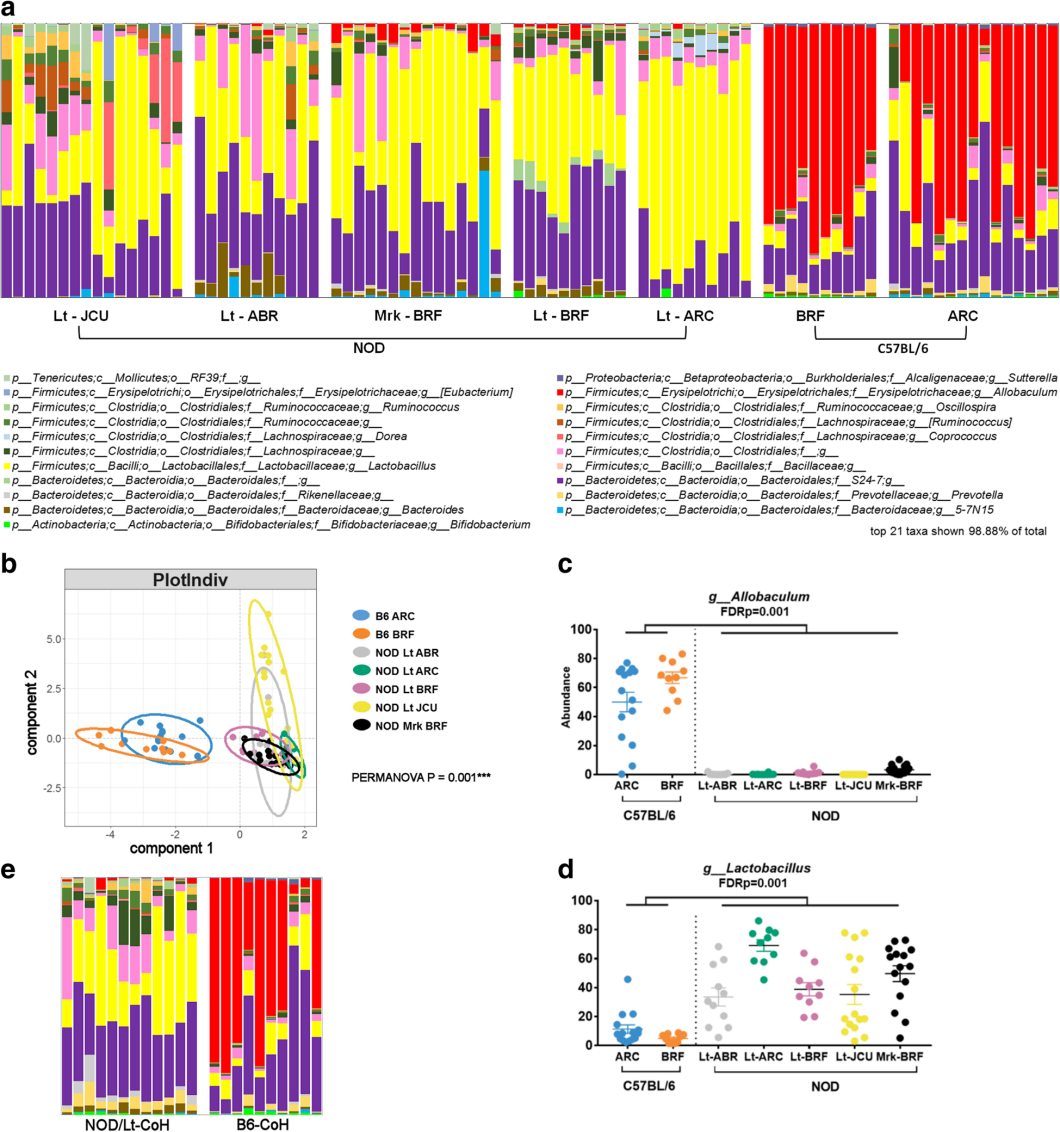
T1D-susceptible NOD mice share similarities in their gut microbiota which is animal facility independent. **a** Stacked bar graphs show top 21 genera present in individual mice. **b** sPLS-DA multivariate analysis. **c** Differences in individual genera, see also Additional file 1: Figure S2. FDR adjusted *p* values are shown from PERMANOVA testing. **d** NOD/Lt and B6 mice (BRF) were cohoused during pregnancy until weaning. Co-housed offspring were sampled at 12 weeks of age for fecal microbiota analysis as in **a**. Control single-strain-housed NOD/Lt and B6 mice (BRF) are shown in **a**. See also Additional file 1: Figures S1 and S3

It is well known that the gut microbiota of mice varies between animal facilities. Despite this, genetic effects dependent on host background can also be observed [19]. To provide evidence that genetics may influence the microbiome in T1D, we sought to determine whether commonalities existed in the gut microbiome of the T1D-susceptible NOD inbred mouse strain independent of the animal facility. To do this, we collected fecal samples from NOD mice bred in four facilities and from two NOD substrains (NOD/Lt and NOD/MrkTac originally derived from Jackson Laboratories and Taconic Farms, respectively). In addition, samples were collected from C57BL/6 (B6) mice bred in two facilities, and microbial profiling was performed by 16S rRNA gene sequencing. Differences in alpha diversity were observable between animal facilities; however, a consistent strain effect was not observed (Additional file 1: Figure S1A, B). A distinct difference between the microbiota composition in mice from the two genetic backgrounds was seen (Fig. 1a). Dimension reduction analysis by supervised sparse partial-least squares discriminant analysis (sPLS-DA) showed substantial overlap of all of the NOD groups which were clearly distinct from the B6 mice (Fig. 1b, PERMANOVA test *p* = 0.001). B6 mice were dominated by members of the genus *Allobaculum* while NOD strains by *Lactobacillus* (Fig. 1c), along with many significant changes in other less abundant taxa (Additional file 1: Figure S2). These data were used to identify seven genera which were the common members of the NOD microbiome present across ≥ 80% of the NOD mice (Additional file 1: Figure S3). By comparison, 15 genera were present in ≥ 80% of the B6 strain (Additional file 1: Figure S3). Although, it is possible that a mismatched sample size (*n* = 62 NOD and *n* = 25 B6) mice contributed to the difference in the number of “core” genera present in ≥ 80% of mice. We concluded that the NOD genetic background is associated with a distinct fecal microbiota to B6 mice.

As differences in the microbiota can be influenced by founder effects driven by caging, litter, or colony drift, we performed a cohousing experiment to test whether the NOD gut microbiota could be substantially altered by early exposure to a different microbiota. We housed pregnant NOD/Lt (BRF) mothers with two B6 females (BRF), which remained with the mother and litter until the pups were weaned. This allowed the pups to be exposed to a B6 microbiota during the critical period of initial colonization after birth. The NOD/Lt-cohoused mice were then separated at weaning and 16s rRNA gene sequencing carried out on fecal samples collected at 12 weeks of age. While we saw an effect of increased alpha diversity in cohoused NOD mice compared with non-cohoused controls (Additional file 1: Figure S1), the microbial profile remained remarkably similar to the non-cohoused NOD/Lt mice (Fig. 1d). The abundance of *Allobaculum*, the dominant genus in the B6 strain, did not significantly increase following cohousing (FDR = 0.706 control NOD vs cohoused NOD). Similarly, the abundance of the seven most dominant genera associated with the NOD genetic background did not significantly change following cohousing (*Lactobacillus* FDR = 0.301, *S24-7* FDR = 0.301, *Clostridiales* FDR = 0.184, *Lachnospiraceae* FDR = 0.523, *Ruminococcaceae* FDR = 0.301, *Oscillospira* FDR = 0.080, and *Ruminococcus* FDR = 0.812 control NOD vs cohoused NOD). We concluded from these data that although the microbiota composition of NOD mice is influenced by housing conditions and early-life exposures to a degree, the overall community composition is constrained by the NOD genetic background.

### NOD mice carrying protective alleles at T1D susceptibility loci *Idd3* and *Idd5* have a distinct microbiota to wildtype NOD mice

**Fig. 2:**
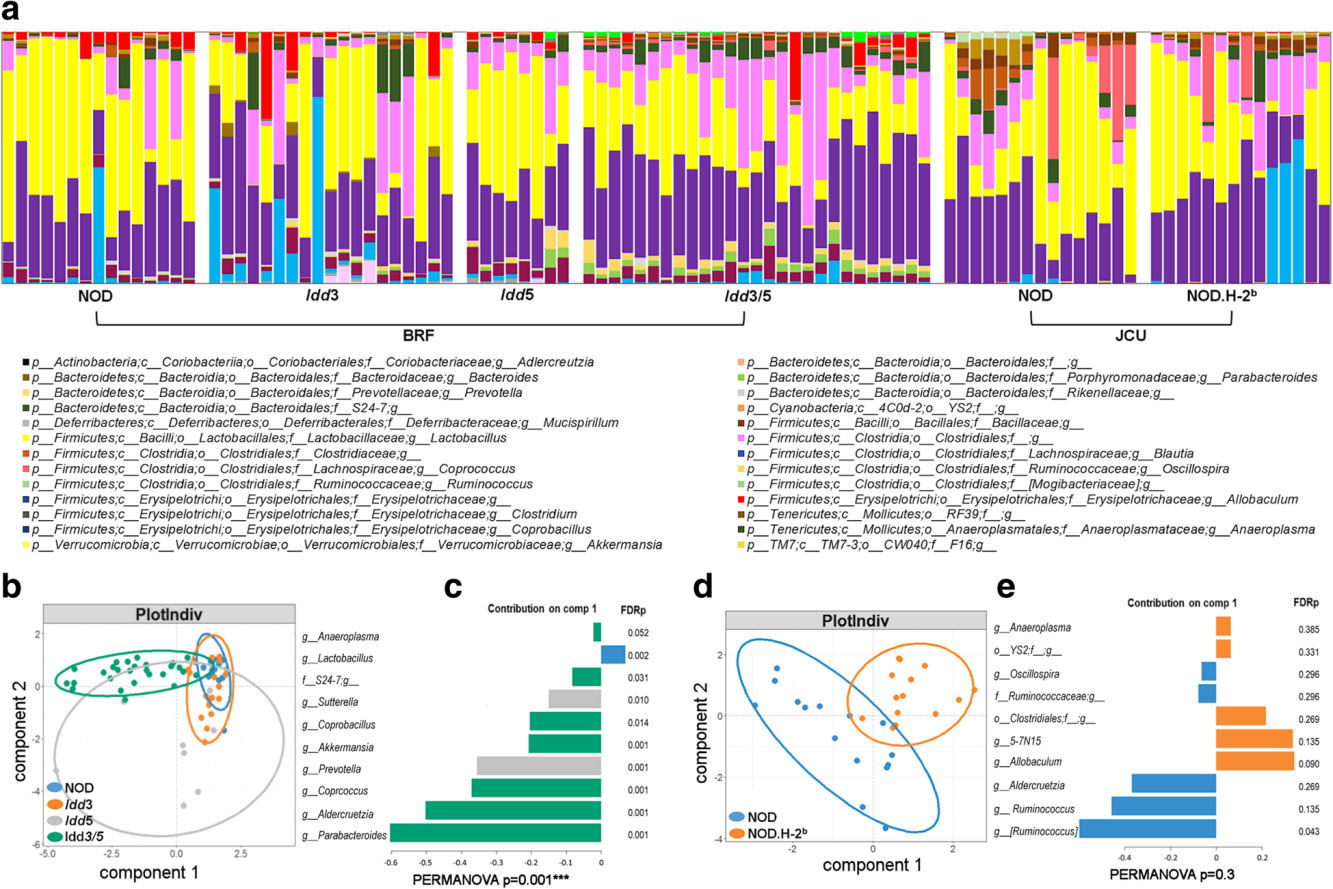
Protective alleles at the *MHC*, *Idd3*, and *Idd5* T1D susceptibility loci are associated with alterations in the microbiota of NOD mice. **a** Stacked bar graphs show top 20 genera present in individual mice. **b**, **d** sPLS-DA multivariate analysis. **c**, **e** Contribution plots showing genera contributing to component 1 of the sPLS-DA plots. *p* values are shown from PERMANOVA testing. FDRp indicates the adjusted *p* value of each genera between strains following PERMANOVA testing. See also Additional file 1: Figures S1, S3, S4, and S5

Our findings so far indicate that the gut microbiota composition of NOD mice from various colonies and housing conditions is restricted by genetic background. While many genetic loci may influence the microbiota profile, we were interested to test whether specific loci known to profoundly impact disease susceptibility contributed to shaping the gut microbiota. To do this, we compared wildtype NOD mice with congenic NOD mice carrying protective alleles at specific T1D susceptibility loci. Congenic NOD.H-2^b^ mice have the NOD H-2^g7^ MHC alleles replaced with H-2^b^ alleles and are completely protected from diabetes development [16]. Likewise, NOD mice carrying protective alleles of both the *Idd3* (*IL2*) and *Idd5* (*Ctla4*, *Slc11a1*, and *Acadl*) loci are also nearly completely protected from disease, whereas mice carrying the individual *Idd3* and *Idd5* regions are partially protected [17, 18]. Microbial profiling of the congenic NOD strains showed that while they still had similarity to the wildtype NOD strains compared to the highly distinct B6 microbiota, *Idd3/5* mice had significant alterations in their microbiota composition (Fig. 2a). sPLS-DA multivariate analysis showed that NOD and *Idd3/5* groups clustered distinctly (Fig. 2b, PERMANOVA *p* = 0.001). The microbiota composition differences included significant alterations in 14 taxa (Additional file 1: Figure S4) with the major drivers of differences between NOD/Mrk and *Idd3/5* mice shown in Fig. 2c. Partially disease-protected *Idd3* and *Idd5* strains had smaller but also significant changes compared with control NOD mice, with *Idd*3 having two and *Idd*5 having ten taxa with FDR < 0.05 (Additional file 1: Figure S4). The *Idd3/5* fecal microbiota had 11 genera common to ≥ 80% of mice with a switch in the relative abundance of *Lactobacillus* and S24-7 compared to NOD along with the addition of *Bacteriodes*, *Parabacteroides*, *Prevotella*, and *5-7N15* (Additional file 1: Figure S3A).

Introduction of protective MHC alleles in NOD.H-2^b^ mice resulted in relatively small overall changes to the microbiota (Fig. 2a). sPLS-DA analysis (Fig. 2d) separated NOD.H-2^b^ and NOD mice with the variance predominately explained by a significant decreased abundance of *Ruminococcus* (FDR = 0.04) and non-significant alterations in a number of other taxa (Additional file 1: Figure S5). The overall difference in variance between NOD and NOD.H-2^b^ mice was not significant by PERMANOVA test (*p* = 0.3). The NOD.H-2^b^ fecal microbiota had six members common to ≥ 80% of mice and was very similar to NOD (Additional file 1: Figure S3). We also compared the common genera from the NOD, B6, *Idd3/5*, and NOD.H-2^b^ strains together by sPLS-DA (Additional file 1: Figure S3B). This clearly showed complete overlap between the NOD and NOD.H-2^b^ while B6 and *Idd3/5* separate from NOD concordantly on component 1, with the drivers shown in Additional file 1: Figure S3C. We concluded that *Idd3/5* risk alleles contributed to maintaining the common gut microbiota features of NOD mice while the MHC alleles had only a minor effect.

### NOD mice have increased inflammation in the ileum and colon compared with disease-protected *Idd3/5*, NOD.H-2^b^, and C57BL/6 mice

**Fig. 3:**
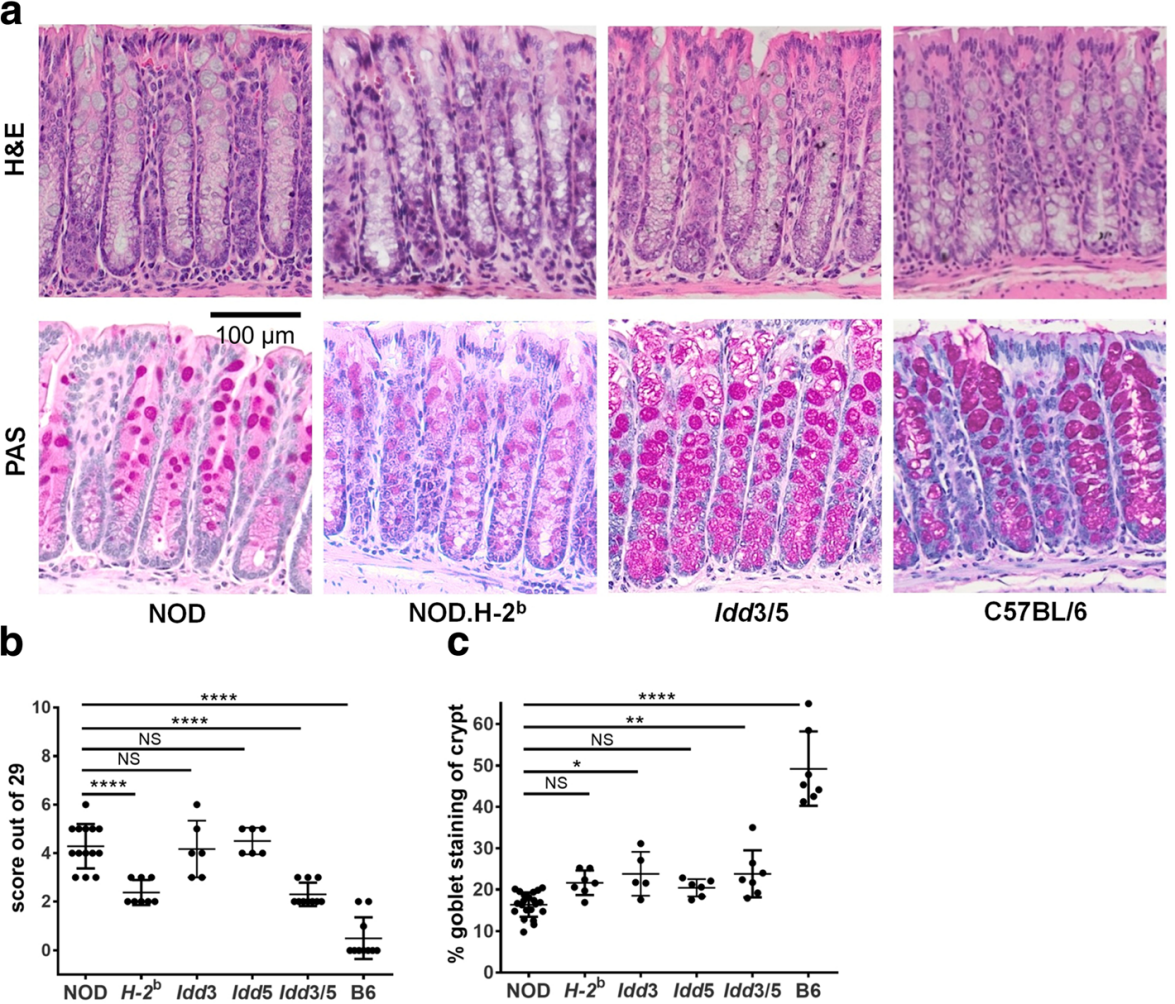
Subclinical inflammation and reduced goblet mucous production in the colon of NOD mice is decreased by protective alleles at the *MHC*, *Idd3*, and *Idd5* T1D susceptibility loci. **a** Representative H&E and PAS-stained sections of the colon. **b** Gut inflammation score for the indicated strains. **c** Mean goblet cell area was calculated from PAS staining of five representative crypts

We hypothesized that the changes we observed in the gut microbiota of disease-protected congenic NOD mice were due to genetically driven changes in the gut immune environment. To this end, we examined histological sections of the ileum, cecum, and colon for signs of inflammation. NOD mice did not have any signs of overt tissue damage or colitis. However, the colon and ileum lamina propria of NOD mice contained an increased frequency of polymorphonuclear leukocytes (PMNs), neutrophils, and lymphocytes compared with disease-protected B6 mice (*p* = 0.0001, Fig. 3a, b). This infiltration was significantly reduced relative to NOD in both NOD.H-2^b^ (*p* = 0.0001) and *Idd3/5* mice (*p* = 0.0001). Partially disease-protected *Idd3* and *Idd5* strains had a similar overall inflammation score to NOD mice (not significant, Fig. 3b). We concluded that autoimmune-susceptible NOD mice have low-level inflammatory infiltration of the colon and ileum.

### Goblet cell mucous production is reduced in NOD mice compared with disease-protected *Idd3/5* and C57BL/6 mice

Goblet cell mucous production is essential for maintaining an effective barrier between the host and gut bacteria [20]. Immune cell-produced regulatory factors such as IL-10 and IL-22 promote mucous production and protect from colitis [21, 22]. The mean area of goblet cell mucous staining was quantified in the colon (Fig. 3a, c). Compared with disease-protected B6 mice, NOD mice had far fewer periodic acid-Schiff (PAS)-stained goblet cells (*p* = 0.0001), and these did not extend as far down the length of the crypt. Although both *Idd3* and *Idd3/5* mice had reduced goblet cell area compared to B6 mice, it was increased compared with NOD mice (*p* = 0.0112 and *p* = 0.0032). Goblet cell area in NOD.H-2^b^ mice and *Idd5* mice was not significantly different to wildtype NOD mice. Therefore, susceptibility alleles at the *Idd3* (IL-2) locus are associated with decreased goblet cell mucous production.

### Paneth cell production of antimicrobial peptides is reduced in NOD mice compared with disease-protected NOD.H-2^b^ and C57BL/6 mice

**Fig. 4:**
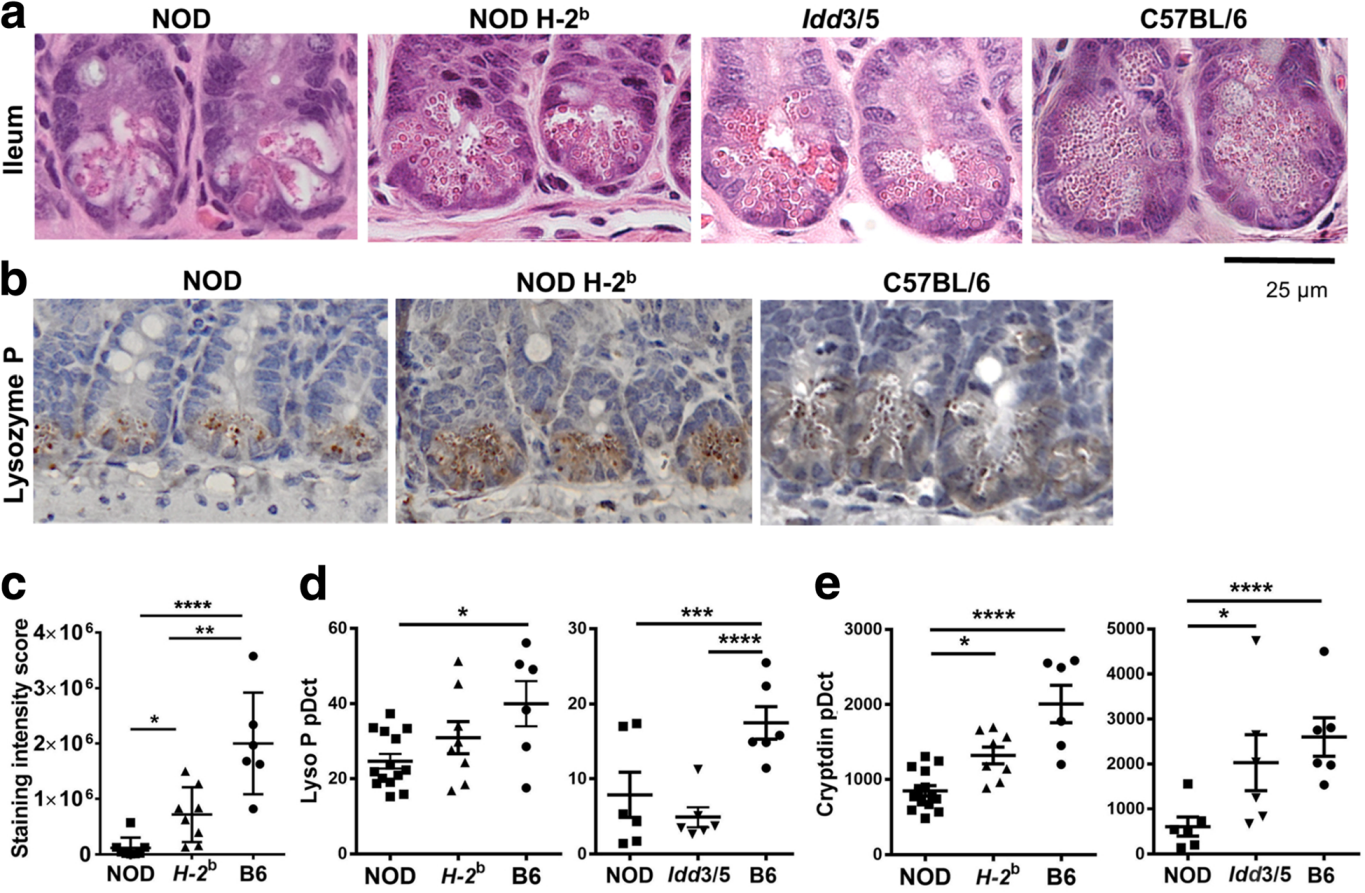
Reduced antimicrobial peptide production from NOD Paneth cells is partly restored by introduction of a protective MHC haplotype. **a** Representative images of H&E stained sections of ileum crypt termini. **b** Lysozyme P staining of crypt termini (brown). **c** Quantification of lysozyme P staining intensity per crypt. Gene expression of *Lysozyme P* (**d**) and *cryptdin* (**e**) relative to *HPRT* in terminal ileum tissue

Compared with B6 mice, Paneth cell morphology in NOD mice was irregular (Fig. 4a). In B6 mice, Paneth cell granules were small, numerous, and densely packed. In contrast, in NOD mice, the granules were large, vacuolar, and fewer in number. *Idd3/5* Paneth cells looked similar to NOD; however, Paneth cell granules in NOD.H-2^b^ mice were smaller and more numerous than NOD. In order to determine whether the production of antimicrobial peptides by Paneth cells was impaired in NOD mice, we stained for lysozyme P (Fig. 4b, c), confirming that Paneth cells were reduced in number and function in NOD mice compared with both NOD.H-2^b^ (*p* = 0.0487) and B6 mice (*p* < 0.0001). *Lysozyme P* and *cryptdin* expression by Paneth cells were also significantly lower in NOD mice compared with B6 mice (*p* = 0.0199 and *p* < 0.0001). NOD.H-2^b^ mice had intermediate *cryptdin* expression (*p* = 0.0061), though *lysozyme P* gene expression did not reach significance (Fig. 4d, e). We concluded that Paneth cell number and function is reduced in NOD mice, and the introduction of protective MHC alleles results in a partial recovery of Paneth cell function.

### Defective regulatory environment within the NOD intestine

**Fig. 5:**
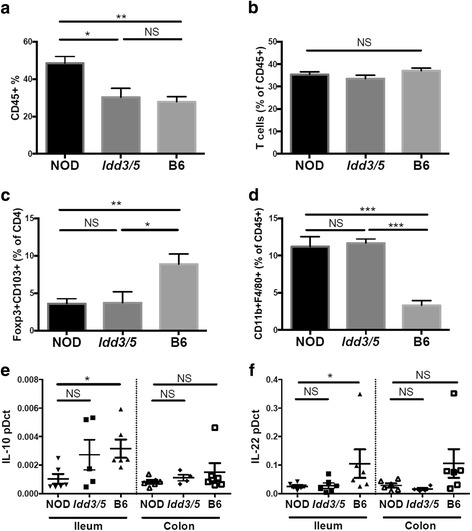
Increased immune cell infiltration and a reduced immunoregulatory environment in NOD intestinal tissue. Colonic lamina propria cells were analyzed by FACS for **a** CD45^+^ cells (*n* = 5–6), **b** CD90^+^ T cells (*n* = 5–7), **c** Foxp3^+^CD103^+^CD4^+^ regulatory T cells (*n* = 5–11), and **d** CD11b^+^F480^+^ macrophages (*n* = 3–6). Gene expression relative to *HPRT* was determined for *IL10* (**e**) and *IL22* (**f**) in the colon and ileum tissue

To investigate the immune cell types infiltrating the NOD colon, we performed FACS analysis of the lamina propria. The NOD colon had a significant increase in the proportion of CD45^+^ immune cells within the lamina propria compared with both B6 (*p* = 0.0065) and *Idd3/5* mice (*p* = 0.0151, Fig. 5a). Within the CD45^+^ population, the proportion of T cells was similar (Fig. 5b). CD103^+^Foxp3^+^ regulatory T cells (Tregs) were significantly enriched within B6 compared with NOD CD4^+^ cells (*p* = 0.0054, Fig. 5c), although CD103^−^ Tregs were not significantly altered (now shown). Within the antigen presenting cell populations, F4/80^+^ inflammatory macrophages were significantly increased in NOD mice compared with B6 (*p* = 0.0002, Fig. 5d). Two key regulatory cytokines that promote immune regulation and gut homeostasis are IL-10 and IL-22. Gene expression of both these cytokines was reduced in the ileum and trended to a decrease in the colon of NOD in comparison with B6 mice (*p* = 0.0268 and *p* = 0.0495, Fig. 5e, f). *Idd3/5* mice had an intermediate level of IL-10 expression but similar IL-22 expression compared with NOD mice. These findings confirmed that NOD mice have increased infiltration of immune cells with an inflammatory rather than a regulatory phenotype in the colon compared with disease-protected B6 mice.

### IL-2 therapy reduces the gut histological inflammation score in NOD mice and alters the gut microbiota

**Fig. 6:**
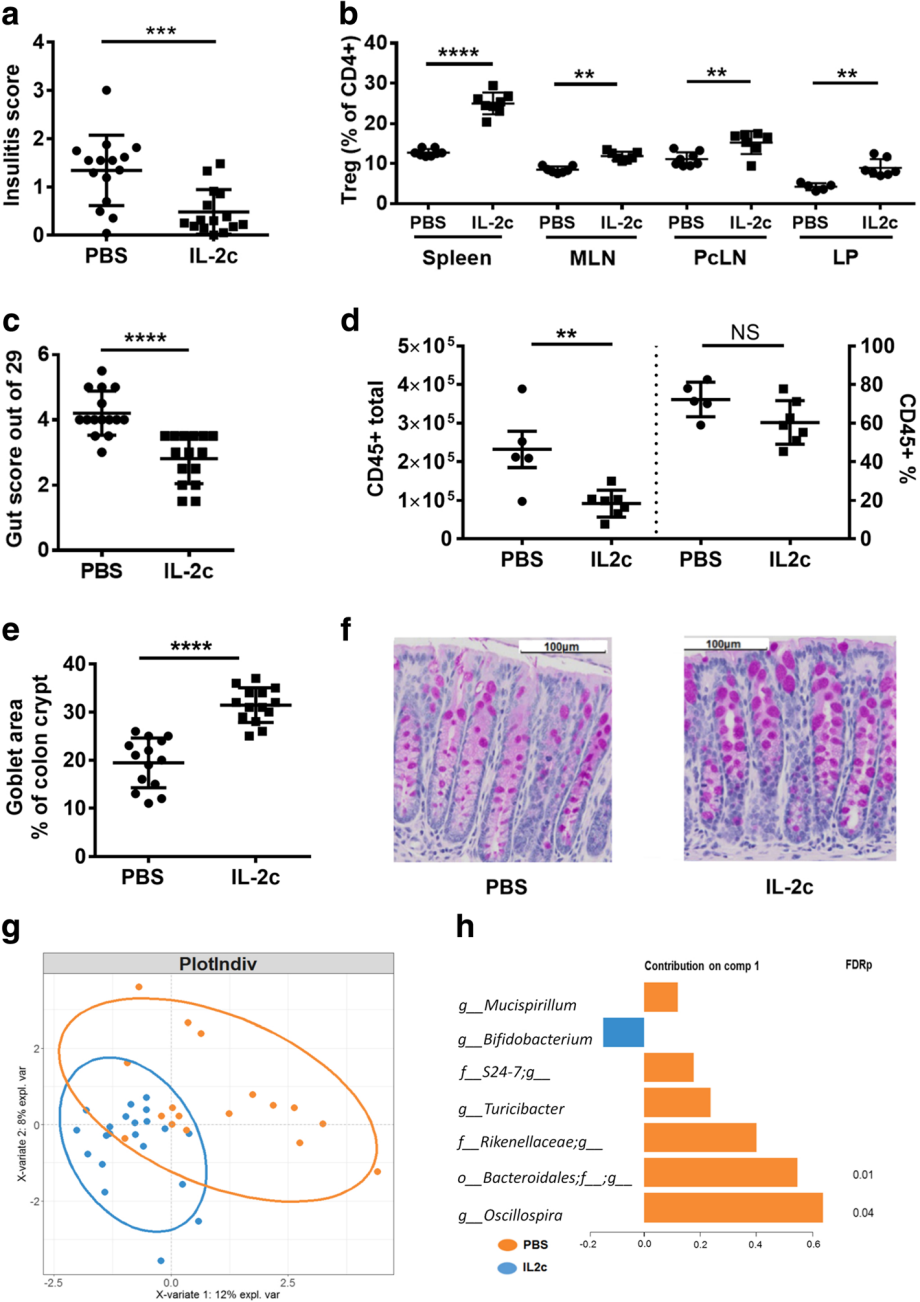
Therapeutic administration of interleukin-2 restores an immunoregulatory environment, reduces gut inflammation, and alters the core gut microbiome of NOD mice. NOD mice were treated with PBS or IL-2c weekly for 6 weeks. **a** Mean insulitis score from the pancreas sections. **b** Foxp3^+^ Treg proportions at the end of treatment in the spleen, mesenteric and pancreatic LN, and colon lamina propria. **c** Overall gut inflammation score. **d** CD45^+^ cell infiltration within the colon lamina propria. **e** Mean goblet cell area was calculated from PAS staining of five representative crypts. **f** Representative PAS-stained section of the colon. Data pooled from two experiments. **g** sPLS-DA multivariate analysis. Data pooled from three experiments. **h** Contribution plot showing genera contributing to component 1 of the sPLS-DA plot. FDRp indicates the adjusted *p* value of each genera between strains following PERMANOVA testing. See also Additional file 1: Figures S1 and S6

NOD alleles at the *Idd3* locus lead to a deficiency in IL-2 production in NOD mice, resulting in defects in Treg function and loss of T cell tolerance [17, 23]. We have previously shown that NOD mice have a generalized reduction in the frequency of Tregs in the lymph nodes, which is contributed to both by *Idd3* and *Idd5* alleles [24]. To test whether long-term treatment with IL-2 would expand Tregs within the gut and reduce inflammation, NOD mice were treated with IL-2c or PBS once a week from 4 to 10 weeks of age. Treatment with IL-2c extends the in vivo half-life of IL-2 and favors expansion of Tregs [25]. In agreement with other reports showing that low-dose IL-2 treatment protects NOD mice from diabetes development, insulitis was significantly reduced in the IL-2c treated mice (*p* < 0.001, Fig. 6a). Treg expansion in the IL-2c-treated mice was confirmed in the spleen, mesenteric LN (MLN), PcLN, and colon lamina propria (*p* < 0.0001, Fig. 6b). Consistent with this, *IL10* but not *IL22* expression was increased in the lamina propria (Additional file 1: Figure S6A). The overall gut inflammation score of the IL-2c-treated mice was significantly reduced compared to PBS-treated mice (*p* < 0.0001, Fig. 6c), which was mainly due to decreased infiltration of PMN cells. Consistent with this, the absolute number of CD45^+^ hematopoietic cells within the colon lamina propria was also decreased (*p* = 0.0075, Fig. 6d). Goblet cell area was also notably increased in IL-2c-treated mice (*p* < 0.0001, Fig. 6e, f). Paneth cell morphology was not altered by IL-2c treatment (Additional file 1: Figure S6B).

In order to determine whether IL-2c treatment also altered the microbiota composition, fecal samples were collected at the end of the treatment period. sPLS-DA analysis showed that IL-2c- and PBS-treated mice had differences in their microbiota composition (Fig. 6g, PERMANOVA *p* = 0.041). The changes in the gut microbiota induced by the IL-2c treatment that was consistent across three experiments were driven by a significant reduction in *Bacteroidales* (FDR = 0.01) and *Oscillospira* (FDR = 0.04) and non-significant decreases in *Rikenellaceae*, *Turicbacter*, *S24-7*, and *Mucispirillum* and an increase in *Bifidobacterium* (Fig. 6h). In one of these experiments, the fecal microbiota was assessed both before treatment (at 4 weeks of age) as well as at the end of treatment (at 10 weeks of age). This analysis demonstrated that the microbiota profile of the two groups was identical before treatment and only diverged following IL-2c treatment (Additional file 1: Figure S6C). Together, these data indicate that IL-2 expands regulatory immune cell populations within the gut tissues, controlling underlying inflammation and influencing the composition of the microbiota.

### T1D-protective alleles linked to the IL-2 signaling pathway are associated with alterations in *Bacteroides*, *Lachnospiraceae*, and *Ruminococcaceae* family members in a healthy human cohort

**Fig. 7:**
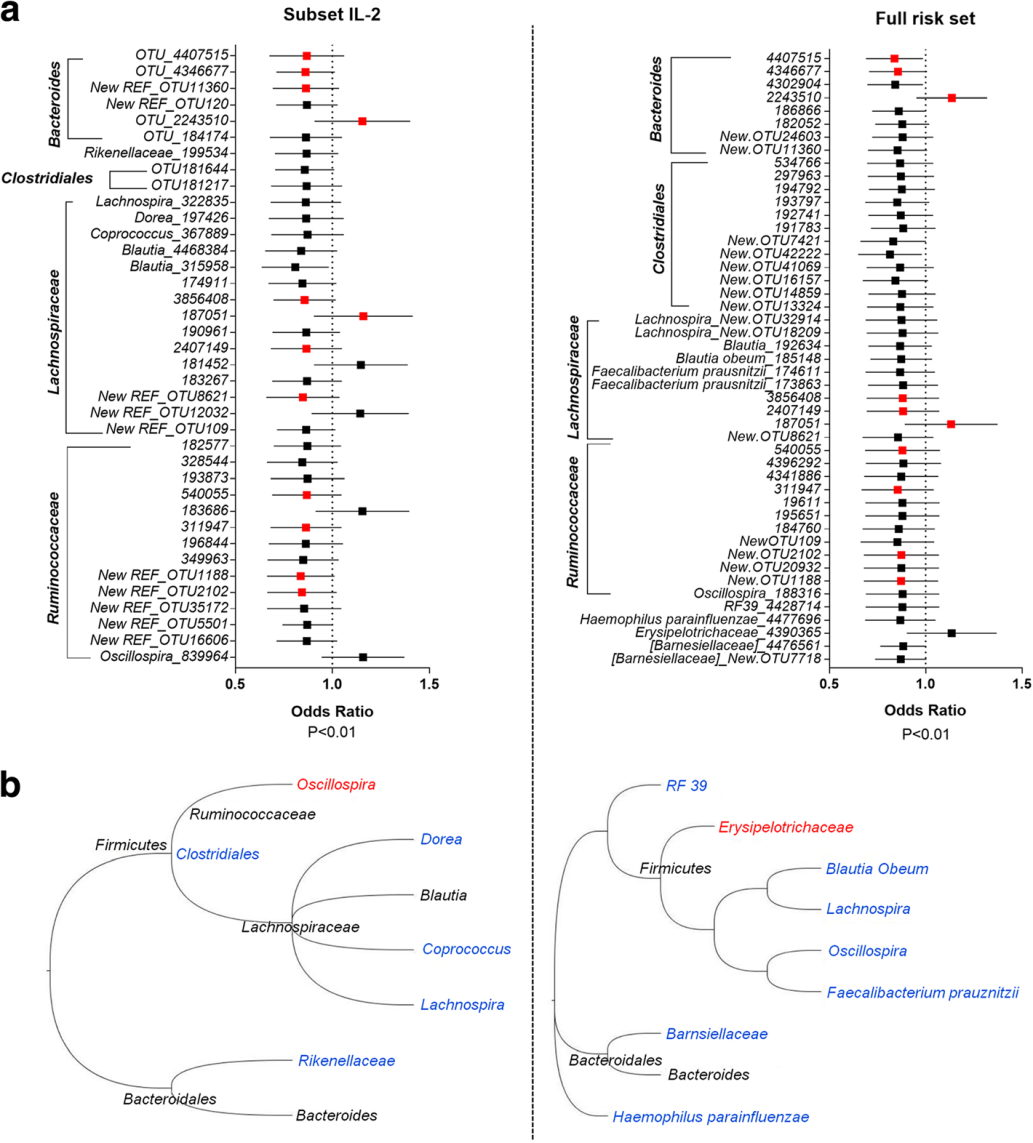
Associations between T1D genetic risk and the microbiota. **a** Each point represents the variance in odds ratio for each OTU with a weighted *p* value of < 0.01 associated with genetic risk of T1D from IL-2/Treg pathway genes (left panel) and all T1D-associated genes (right panel). Bars represent the standard error. Red points indicate OTU shared between left and right panels. **b** Phylogenetic trees of the OTU identified in **a**. Red indicates increased with T1D risk, blue indicates decrease with T1D risk, and black indicates both increases and decreases observed. IL-2/Treg pathway genes (left panel) and all T1D-associated genes (right panel)

To explore whether similar effects to those we observed from *Idd3/5* alleles on the gut microbiota could be mediated by T1D susceptibility alleles in humans, we utilized a cohort of healthy human samples from the TwinsUK study [26]. This study included matched stool microbiome and SNP genotyping data from 1392 individuals. We looked for associations between specific taxa and genotype at a number of T1D-associated SNPs linked to the IL-2 signaling pathway and Treg function*.* A combined genetic risk score was developed for SNPs in IL-2 interacting genes. The genes included were selected from IL-2 interacting genes determined by STRING network analysis (*IL2*, *IL2RA*, *IL10*, *IL27*, *IL7R*, and *DGKA* region SNPs); *CTLA4* was included due to its overlap with *Idd5* and role in Treg function and *PTPN2* due to the described effects of T1D-associated *PTPN2* SNPs on IL-2 signaling [27]. A combined risk score involving all SNPs within these genes was calculated for each subject based on their genotype at these loci and weighted using the odds ratio of each loci. The weighted risk score was then used to perform a linear regression to determine associations with the operational taxonomic unit (OTU) identified in these individuals. Although we were underpowered to identify significant associations after adjusting for false discovery rate, 38 OTUs were identified with an unadjusted *p* value < 0.01 (Fig. 7a, b and Additional file 2: Table S1). This analysis suggests that susceptibility T1D alleles within the IL-2/Treg pathway may be associated with a decreased abundance of a number of members of the *Clostridiales*, *Bacteroides*, *Lachnospiraceae*, *Ruminococcaceae*, and *Rikenellaceae* families, while only a few OTUs were positively associated with T1D risk. These associations overlapped with the effects of *Idd3/5* alleles in the NOD mouse, with *Lachnospiraceae* members including *Coprococcus*, *Bacteroides*, and unclassified *Clostridiales* associated with protection. We then repeated this analysis using a risk score calculated from all known T1D-associated SNPs. This analysis found very similar associations with a decreased abundance of members of the *Clostridiales*, *Bacteroides*, *Lachnospiraceae*, and *Ruminococcaceae* families associated with disease risk including *Faecalibacterium prausnitzii*, which has known protective associations in other autoimmune diseases (Fig. 7c, d and Additional file 3: Table S2). These data indicate that protective alleles at T1D-associated risk loci may modify the composition of the gut microbiota in humans.

## Discussion

Colonization of the gastrointestinal tract soon after birth is determined by a complex interplay between environmental modifiers and the host metabolic state and intestinal immune response, factors significantly affected by host genetics. Human twin studies and studies of intercrossed mice bred in highly controlled environments have enabled investigations into the contribution of host genetic factors in shaping the gut microbiota (reviewed in [28]). These studies have identified genes involved in metabolic function and innate immune activation and regulation. Our findings that protective *Idd3/5* alleles, which are associated with enhanced IL-2 production and regulatory T cell function, give rise to notable alterations in the microbiota fit well with the premise that regulatory immune cell populations of the lamina propria are able to modulate the gut microbial community [29].

Recently, genome-wide association studies (GWASs) of the gut microbiota in humans have also attempted to identify genetic loci or pathways associated with changes in the abundance of individual microbial taxa [26, 30–32]. Although these studies all concluded that host genetic factors did contribute to the variation in the microbiota, effect sizes of individual loci were small and varied between studies. The loci identified included SNPs in immune response genes linked to autoimmune diseases including Crohn’s disease, ulcerative colitis, multiple sclerosis, and T1D (*CD5*, *CCL2*, *IL23R*, *CD86*, *HLA*, *C11ORF30-LRRC32*, *CLEC16A*) [30–32]. Rather than looking for associations with individual SNPs, we performed a pathway analysis to look for associations with genes linked to IL-2 signaling and Treg function and the microbiota composition. Suggestive associations were observed between a low disease risk score and the abundance of members of the *Clostridiales*, *Bacteroides*, *Lachnospiraceae*, and *Ruminococcaceae* families. Although our analysis utilized data from 1392 individuals, a substantially larger GWAS scale dataset will be needed to provide sufficient statistical power to confirm these findings. Nevertheless, our study supports a role for natural variation in immune response genes influencing the gut microbiota, although high variability between populations originating from diverse geographical locations clearly is an impediment to the replicability of these findings.

GWAS studies of the microbiota in both mouse and humans have shown that certain taxa have a higher heritability than others [26]. This may be due to the relative sensitivity or lack of adaptability of these bacteria to changes in their preferred environment. For example, *Akkermansia*, which is associated with the mucin layer, was associated with *SIGLEC15* gene expression [26]. *SIGLEC15* is associated with expression of a carbohydrate found bound to mucin. *Akkermansia*, which was increased in *Idd3/5* mice, has been associated with a healthy microbiota via improved epithelial integrity [33]. Other mucinophiles such as *Bacteroidales* members including *Parabacteriodes* were significantly increased in protected *Idd3/5* mice. *Parabacteriodes distasonis* abundance was decreased in inflamed tissue biopsies of IBD patients [34], and *P. distasonis* administration reduced the severity of DSS-induced colitis suggesting a protective role in the colon [35]. Another mucin utilizer, *Aldercruetzia* from the *Actinobacteria* phyla, was also increased in *Idd*3/5 mice. *Aldercruetzia* has been associated with health as a reduction in *Aldercruetzia* was observed in patients with multiple sclerosis compared to healthy individuals [36]. The increase in mucinophiles observed in *Idd3/5* mice may be related to the increase in goblet cell mucous production observed in this strain.

Taxa that have been previously reported as heritable include many of those found with differential abundance between either the NOD, *Idd3/5*, or B6 core microbiomes for example *Lactobacillus* [32], *S24-7* [37], *Ruminoccoccus* [31], *Oscillospira* [37], *Bacteriodes* [38], *Ruminococcaceae* [26, 32, 37], *Lachnospiraceae*, [37], and *Prevotella* [31]*.* Several of these taxa also had a suggestive association with T1D genetic risk in our human association study (e.g., *Ruminococcaceae*, *Lachnospiraceae*, *Bacteroides*, and *Oscillospira*). This supports our findings that the NOD microbiome is maintained by host genetics.

Several studies have investigated alterations in the microbiota in human subjects with a high genetic risk of developing T1D either with or without islet autoimmunity (defined by the presence of autoantibodies) or in longitudinal study designs monitoring individuals before and after disease onset [5–7]. Although differences have been found between these studies, which could be related to sample size, differences in study design, and geographic locations, several interesting findings have been noted. Kostic et al. performed a longitudinal study of a Finnish cohort and found that alpha diversity dropped in T1D cases after seroconversion but prior to clinical diagnosis of disease [6]. This was driven by a relative overabundance of several groups including *Ruminococcus*, *Blautia*, and *Streptococcus* while there was a relative underabundance of *Veillonellaceae* and *Lachnospiraceae* in T1D converters. Consistent with this, *Lachnospiraceae* was reduced in the *NOD* compared with the *Idd3/5* core microbiome and also associated with protective genotypes in the human analysis. In a cross-sectional study, Alkanani and colleagues found a trend towards a reduced abundance of the genera *Prevotella* in individuals with a higher risk of disease progression [7], and increased *Prevotella* in healthy children compared to those with T1D was seen in another study [39]. These findings are similar to the increase in *Prevotella* that we found in *Idd5* and *Idd3/5* mice compared with NOD mice. One early study found an increase in *Faecalibacterium prausnitzii* in control children compared with those with islet autoimmunity [4]. *F.prausnitzii* is a butyrate producer associated with gut health that is depleted in Crohn’s disease [40] that was associated with a T1D-protective genotype in our risk set analysis. While *F.prausnitzii* are not members of the mouse gut microbiome, other butyrate-producing bacteria such as *Coprococcus* from the *Lachnospiraceae* family fill a similar niche [41], and abundances of these were increased significantly in *Idd*3/5 compared with NOD mice. Thus, although there are major differences between the murine and human microbiota, some commonalities emerge, suggesting that both in mice and humans, changes in some T1D-associated microbes are caused by host immunogenetic factors rather than just the disease itself.

Treatment with IL-2 resulted in highly significant effects on the intestinal phenotype including reduced immune infiltration and increased goblet cell mucous production. This was associated with alterations in the microbiota including decreased *Oscillospira* and *Bacteriodales*. Although *Oscillospira* was not significantly altered by *Idd3* or *Idd3/5* in comparison to control NOD/Mrk mice, the overall abundance of *Oscillospira* in the combined NOD colonies was significantly higher than both the *Idd3/5* and B6 cohorts (Additional file 1: Figure S3). Similarly, *Oscillospira_839964* trended to be increased by the human IL-2 risk alleles, suggesting a common IL-2-dependent mechanism may drive down *Oscillospira* abundance in humans and mice. In contrast, *Bacteriodales* was reduced by IL-2c treatment but was increased in *Idd3* mice although remained low in *Idd3/5* mice. This suggests that *Bacteriodales* abundance is influenced by other factors in addition to IL-2.

Despite being profoundly protected from T1D, we found only subtle changes in the fecal microbiota of NOD.H-2^b^ mice in comparison with control NOD mice with only *Lachnospiraceae* [*Ruminococcus*] significantly different after adjustment for multiple testing. This was despite a reduction in immune infiltration in the colon and ileum of NOD.H-2^b^ mice and improved Paneth cell morphology. This suggests that the phenotypic differences found between NOD and NOD.H-2^b^ guts were primarily driven by host genetics although feedback from the microbiota may exacerbate them. A recent study described differences in the cecal microbiota between NOD mice transgenic for the MHC-II Eα gene and control NOD mice [42]. Wildtype NOD but not B6 mice lack the E complex gene, and Eα16/NOD transgenic mice are highly protected from diabetes. Silverman and colleagues found differences in the overall diversity of the microbiota between these strains although only the order *Clostridales* and the genus *Blautia* were individually significant. As this study sampled the cecal contents rather than the feces, it is possible that the impact of the Eα gene was greater in the cecum than in the colon. In line with this, the only immune cell difference detected in Eα16/NOD mice was in Treg abundance within the cecum but not the small or large intestine.

Our analysis of immune cell populations found within the NOD intestine pointed to an increase in inflammatory populations. Although neither NOD mice nor patients with T1D typically present with the overt gut disease, previous studies have reported intestinal inflammation or immune activation using biopsy tissue from the duodenum of T1D patients. In comparison with healthy individuals, T1D patients had increased HLA-class II, IL-1α, and IL-4 expression in the jejunum [43]. Additionally, T1D patients had significantly fewer Foxp3^+^ Tregs in the duodenum, and lamina propria dendritic cells isolated from the duodenum were not able to induce Treg differentiation [44]. NOD mice were reported to have increased proportions of T cells with an activated phenotype and producing IL-17 in the colon [45]. Together with our findings, these data suggest that increased intestinal immune cell activation and infiltration are associated with T1D and are likely to at least partly underlie changes in the microbiota associated with T1D.

Paneth cell dysfunction is associated with Crohn’s disease and is linked to the presence of risk alleles for an autophagy gene *ATG16L1* [46]. Paneth cells produce antimicrobial peptides, which are released into the crypt lumen where they protect against infection by enteric pathogens and help to shape the commensal microbiota. Paneth cell degranulation coupled with the death of the Paneth cells can be induced by IFNγ exposure [47, 48]. Paneth cell dysfunction can also be induced by unfolded protein response-induced ER stress [49], which can be exacerbated by IL-10 deficiency [21]. As such, a combination of increased infiltration of inflammatory cells along with impaired regulatory T cell responses may contribute to Paneth cell dysfunction in NOD mice.

Another factor known to influence the composition of the microbiota in NOD mice is gender [50, 51]. In this case, testosterone produced in males is thought to be metabolized by certain bacterial taxa influencing their activity and community composition. This results in a feedback loop whereby androgen levels influence the microbiota which impact inflammation and the immune response giving rise to disease protection in males. This is therefore another example whereby host genetics (male sex) influences the microbiota and diabetes incidence.

One limitation of our analyses was the relatively low sequencing depth used after rarefaction of the data. Rarefaction is a useful means of normalizing microbiome data to account for varied ability to detect rare taxa depending on sequencing depth. However, this will have limited our ability to detect significant alterations in low abundance taxa. Another limitation of our cohousing study was that the co-housed NOD pups were still nursed by their own NOD mothers despite the presence of B6 females within their cages. The lack of B6-derived maternal factors (e.g., maternal antibodies) passed to the NOD pups may have reduced the efficiency of colonization by B6-derived taxa as described by others [52, 53]. We also note that our studies of the microbiota of the various congenic NOD strains were not controlled for with non-congenic littermate animals. This would additionally control for differences in exposure to various microbial taxa due to founder effects that may have occurred when each line was established.

## Conclusions

In conclusion, we show that natural variation in genetic loci associated with risk of T1D influence the structure of the gut microbiota. Pathways linked to IL-2 signaling and immune regulation were associated with microbial shifts in both mouse and human with higher disease risk associated with a decrease in the presence of *Ruminococcus*, *Lachnospiraceae*, and *Clostridiales*. These taxa have previously been reported to have a high heritability estimate in human and mouse studies. Mechanistically, we found that the IL-2 pathway profoundly impacted inflammation present in the colon and small intestine with IL-2 therapy increasing Tregs and immunoregulatory cytokines promoting increased goblet cell mucous production. This suggests that immunotherapy strategies currently aimed at supplementing IL-2 and boosting Treg function in T1D patients may potentially influence the microbiota in these patients and microbiota monitoring strategies could be investigated for determining response to therapy.

## Methods

### Mice

Female non-diabetic mice were housed in SPF conditions at the Biological Resource Facility (BRF) of the Translational Research Institute (TRI). NOD/Lt (Jackson substrain NOD mice) and B6 mice were purchased from the Animal Resources Centre (ARC, Perth, WA, Australia). NOD/Lt and C57BL/6 mice originally purchased from ARC were also bred in the BRF and used for analysis as indicated. Additional NOD/Lt mice were purchased from Australian BioResources (ABR, Moss Vale, NSW, Australia). NOD.H-2^b^ mice have the NOD MHC replaced with the MHC derived from C57BL/10 mice and is highly protected from disease [16] were a gift from Prof. Alan Baxter, (James Cook University [JCU], QLD, Australia). NOD.H-2^b^ mice were compared with NOD/Lt mice also from JCU. NOD/Mrk (Taconic substrain NOD mice) *Idd3*, *Idd5*, and *Idd3/5* disease-protected congenic NOD mice were originally purchased from Taconic Farms and have been described previously [17, 18, 23]. *Idd3* mice (Taconic strain 1098) carry a 2.7-Mb genetic interval on chromosome 3 derived from the C57BL/6 strain. *Idd5* mice (Taconic strain 1094) carry a 28.3-Mb genetic interval on chromosome 1 derived from the C57BL/10 strain. *Idd3/5* mice (Taconic strain 1591) contain both intervals. Mice from ARC, ABR, and JCU were shipped by air to the University of Queensland BRF facility and the fecal microbiota samples collected within 1 week of arrival.

### 16S rRNA gene sequencing

Fresh fecal samples were collected from 9- to 12-week-old female mice derived from at least two different cages and frozen on dry ice before storage at − 80 °C. Bacterial DNA was extracted from frozen fecal pellets using the automated Maxwell 16 Research System (Promega) with Blood LEV kit (Promega) following the manufacturer’s instructions. An adapted 16S rRNA gene Metagenomic Sequencing Library Preparation protocol (Illumina) was utilized to amplify the 16S rRNA gene V6-V8 (926-1392) region. A first-step amplicon PCR was conducted to amplify the V6-V8 region with Illumina-specified, region-specific primers (forward AAACTYAAAKGAATTGACGG, primer ACGGGCGGTGTGRC, Integrated DNA Technologies, NSW, Australia) using Q5 High-Fidelity DNA Polymerase (New England BioLabs, Inc.; Ipswich, MA, USA). PCR products were visualized and cleaned up using the E-Gel Electrophoresis System with 2% SizeSelect gels (Life Technologies; Carlsbad, CA, USA). A second-step indexing PCR was then used to attach indices (Illumina Nextera XT Index Kit v2) to each sample. Following PCR clean up using AMPure XP beads (Beckman Coulter, Inc.; Brea, CA, USA), each sample was normalized to equimolar concentrations of DNA. The pooled PCR product was sequenced and the reads demultiplexed by the Australian Centre for Ecogenomics (ACE) (Brisbane, QLD, Australia) on an Illumina MiSeq platform. A median of 15,135 reads per sample was obtained. Sequences were analyzed using QIIME software (MacQIIME_1.9.1-OS10.7) using closed reference picking and Greengenes bacterial database version 13_8 (The Greengenes Database Consortium; http://greengenes.secondgenome.com) with 97% similarity to cluster the operational taxonomic units (OTUs) in each sample. Samples were filtered to exclude sequences that were not archaea or bacteria, then filtered to remove low abundance OTUs set at 0.01%. BIOM tables were exported in tab-delimited form and information summarized with taxonomy at genus level. Rarefaction was applied in 1769 after viewing the diversity metrics. Communities were visualized with R package mixOmics for sparse partial-least squares discriminant analysis (sPLS-DA) and for visualizing plot loadings [54]. Ellipses represent the 90% confidence interval.

### Quantitative real-time PCR

Intestinal tissues were collected and immediately stored in RNALater (Life Technologies). After removal of the RNALater, the tissues were homogenized using Trizol (Life Technologies) and 1.0-mm silicon carbide beads (Biospec Products) on the Precellys 24 at 4000 rpm for 30 s, three times. After centrifugation, the supernatant was collected and RNA extraction performed using a Direct-zol RNA purification kit (Zymo Research). cDNA was synthesized from tissue RNA (Sensifast cDNA synthesis kit, Bioline) and real-time PCR used the SensiFast SYBR Lo-ROX Kit (Bioline) on a ViiA7 real-time PCR system (Life Technologies). Data were analyzed in duplicates using the delta-delta Ct method, with HPRT as the housekeeping gene. Primers used are shown in Table 1.

**Table 1:** Primers for qRT-PCR

IL-10	′5-CAGGGATCTTAGCTAACGGAAA-3′
	′5-GCTCAGTGAATAAATAGAATGGGAAC-3′
IL-22	′5-ATGAGTTTTTCCCTTATGGGGAC-3′
	′5-GCTGGAAGTTGGACACCTCAA-3′
HPRT	′5-CCTAAGATGAGCGCAAGTTGAA-3′
	′5-CCACAGGACTAGAACACCTGCTAA-3′
Lysoyzme P	′5-ATGGCTACCGTGGTGTCA-3′
	′5-CGGTCTCCACGGTTGTAGTT-3′
Cryptdin	′5-AAGAGACTAAAACTGAGGAGCAGC-3′
	′5-GGTGATCATGAGACCCCAGCATCAGT-3′

### Histology

Histology sections were prepared from rolled gut tissue and stained with hematoxylin and eosin (H&E) and periodic acid-Schiff stain (PAS). Lysozyme staining was performed using rabbit anti-lysozyme antibody (Dako, USA) and secondary HRP-conjugated anti-rabbit antibody (Thermo Fisher). Staining was visualized using DAB. Scoring was employed to examine the integrity of the pancreas, colon, and ileum in a blinded manner. Parameters examined were Paneth cell morphology within the ileum, crypt architecture within the colon, and inflammatory cell infiltration in the lamina propria (Table 2) based on [21]. Goblet cells were measured by calculating the area of PAS staining as a percentage of total crypt area (average five crypts) within the medial colon region. Insulitis was scored in at least 20 islets per mouse as either 0, no infiltration; 1, peri-insulitis; 2, mild-invasive insulitis; or 3, severe invasive insulitis [23].

**Table 2:** Gut scoring matrix (maximum 29)

Feature	Score	Description
Colon		
Crypt architecture	0	Normal
	1	Irregular
	2	Moderate crypt loss (10–50%)
	3	Severe crypt loss (50–90%)
	4	Small/medium ulcers (< 10 crypt widths)
	5	Large ulcers (> 10 crypt widths)
Crypt abscesses	0	None
	1	1–5
	2	6–10
	3	> 10
Crypt length (medial colon)	0	< 250 μm
	1	250–300 μm
	2	300–350 μm
	3	350–400 μm
	4	> 400 μm
Tissue damage	0	No damage
	1	Discrete lesions
	2	Mucosal erosions
	3	Extensive mucosal damage
Inflammatory cell infiltration	0	Occasional infiltration
	1	Increasing leukocytes in lamina propria
	2	Confluence of leukocytes extending to submucosa
	3	Transmural extension of inflammatory infiltrates
Lamina propria neutrophils (PMN)	0	0–5 PMNs/HPF
	1	6–10 PMNs/HPF
	2	11–20 PMNs/HPF
	3	> 20 PMNs/HPF
Ileum		
Inflammatory cell infiltration	0	Occasional infiltration
	1	Increasing leukocytes in lamina propria
	2	Confluence of leukocytes extending to submucosa
	3	Transmural extension of inflammatory infiltrates
Lamina propria neutrophils (PMN)	0	0–5 PMNs/HPF
	1	6–10 PMNs/HPF
	2	11–20 PMNs/HPF
	3	> 20 PMNs/HPF
Paneth cell	0	Normal: small, densely packed granules
	1	Irregular: less granules
	2	Abnormal: less granules, large vacuolar spaces

### Mouse treatments

IL-2/anti-IL-2 complexes (IL2c) were given by IP injection of complexes containing 1 μg IL-2 (BioLegend, San Diego, CA) and 5 μg anti-IL-2 (JES6-1, Bio X Cell, West Lebanon NH).

### Lamina propria cell preparation

Laminar propria lymphoid cells were prepared from sectioned intestinal tissue washed with cold PBS then stirred at 37 °C for 20 min in Hanks’ Balanced Salt Solution (HBSS) containing 5% FCS and 3 mM EDTA. The intraepithelial lymphocytes (IELs) in the supernatant were collected and washed with RPMI 1640. Intestine pieces were chopped into fine pieces and stirred in RPMI 1640 containing 10% FCS, collagenase D (1 mg/ml; Roche), and DNase I (40 μg/ml; Sigma-Aldrich) at 37 °C for 40 min. The lamina propria lymphocytes in the supernatant were collected and washed with RPMI.

### Flow cytometry

Single cell suspensions were stained using a live-dead staining kit from Biolegend (San Diego, CA). Antibodies used were CD3 (17A2), CD4 (GK 1.5), CD8 (53-6.7), CD11c (N418), CD19 (6D5), CD25 (PC61), CD90.2 (30-H12) from Biolegend (San Diego, CA) and CD45.1 (A20), CD45.2 (104), and Foxp3 (FJK-16S) from eBioscience (San Diego, CA). Foxp3 was detected using the Foxp3 Staining Set (eBioscience). Samples were run on an LSRFortessa X-20 (BD Bioscience) and analyzed using FlowJo (Tree Star).

### T1D risk score association analysis

IL-2 pathway-associated SNPs were determined using the STRING database (http://string-db.org) to identify T1D candidate genes linked to IL-2 or IL-2RA with evidence of association either experimentally determined, from curated databases or by co-expression. Matched genotype and OTU abundance from 16S rRNA gene sequencing data were obtained from TwinsUK registry in 1392 otherwise healthy individuals. Genotyping, 16S rRNA amplicon sequencing, filtering, and analysis were performed as described [55].

T1D-associated SNPs used in calculating the risk scores are listed in Additional file 4: Table S3. The risk score was weighted by risk allele dosage, and effect size was reported in the relevant published papers and databases (Additional file 4: Table S3), then the dosages summed. Allelic risk scores were calculated using custom scripts in R 3.3.3 [56]. Linear mixed effects models were used to investigate the association between each of the OTUs and the 112 individual T1D SNPs or allelic score. These models account for the correlation between twins within a family. Analysis of the single T1D-associated SNP was conducted in Merlin [57]. This uses a likelihood ratio test to compare a model including the individual SNP to a model without the SNP. Descriptive statistics and the analysis of the allelic score were conducted in R (version 3.3.3) using the nlme library [56].

### Statistical analysis

Statistical analyses were performed with GraphPad Prism Software (version 7; GraphPad Software, Inc.). Mean and SEM are shown unless otherwise stated. Statistical significance was reached when *p* ≤ 0.05. Significance denoted *p ≤ 0.05, **p ≤ 0.01, ***p ≤ 0.001, and ****p ≤ 0.0001. For one way ANOVA, either the non-parametric Kruskal-Wallis test was used, and correction for multiple testing either used Dunn’s or the Benjimi-Hochberg method or Tukey’s multiple comparison test was used for normally distributed data. For FDR multiple testing of microbiota abundance, we used the R package “RVAideMemoire” and “Vegan” for the PERMANOVA and anosim functions with randomization set at 4000.

## Additional files

Additional file 1: Supplemental information. (PDF 5693 kb)

Additional file 2: Table S1. OTU associations with IL-2/Treg pathway risk alleles. Related to Fig. 7. (XLSX 67 kb)

Additional file 3: Table S2. OTU associations with all T1D-associated risk alleles. Related to Fig. 7. (XLSX 61 kb)

Additional file 4: Table S3. T1D-associated SNPs used for risk score calculation. Related to Fig. 7. (XLSX 49 kb)

## Abbreviations

ABR: Australian BioResources
ARC: Animal Resources Centre
B6: C57BL/6
BRF: Biological Resources Facility
FDR: False discovery rate
*Idd*: Insulin-dependent diabetes
IL-2: Interleukin-2
JCU: James Cook University
MHC: Major histocompatibility complex
MLN: Mesenteric lymph node
NOD: Non-obese diabetic
PcLN: Pancreatic lymph node
PMN: Polymorphonuclear leukocytes
sPLS-DA: Sparse partial-least squares discriminant analysis
T1D: Type 1 diabetes
Treg: Regulatory T cell
