# Correction to: Type 1 diabetes susceptibility alleles are associated with distinct alterations in the gut microbiota

**DOI:** 10.1186/s40168-018-0438-z

**Published:** 2018-03-20

**Authors:** Jane A. Mullaney, Juliette E. Stephens, Mary-Ellen Costello, Cai Fong, Brooke E. Geeling, Patrick G. Gavin, Casey M. Wright, Timothy D. Spector, Matthew A. Brown, Emma E. Hamilton-Williams

**Affiliations:** 1 Translational Research Institute, The University of Queensland Diamantina Institute, University of Queensland, QLD, Brisbane, Australia; 2 AgResearch Limited, Grasslands Research Centre, Palmerston North, New Zealand; 3 Institute of Health and Biomedical Innovation, Queensland University of Technology, Translational Research Institute, QLD, Brisbane, Australia; 4 Department of Twin Research and Genetic Epidemiology, King’s College London, London, SE1 7EH UK; 5 Translational Research Institute, The University of Queensland Diamantina Institute, 37 Kent St, Woolloongabba, QLD 4102 Australia

## Correction

Following publication of the original article [1] it came to the attention of the Production Editor that Figs. 1 and 2 had not been replaced with the newly revised figures supplied by the authors (the originals being unusable due to poor quality image and text).

The original article has been corrected.
