# Supplementary material for: Type 1 diabetes susceptibility alleles are associated with distinct alterations in the gut microbiota
Source: Microbiome. 2018 Feb 17;6:35. doi: 10.1186/s40168-018-0417-4 (PMC5816355; doi:10.1186/s40168-018-0417-4)
Supplement: Supplementary file 1 — Supplemental information. (PDF 5693 kb) [file 40168_2018_417_MOESM1_ESM.pdf]

Supplemental Information

Type 1 diabetes associated risk alleles contribute to increasing intestinal inflammation and altered microbiota composition

Jane A. Mullaney, Juliette E. Stephens, Mary-Ellen Costello, Cai Fong, Brooke Geeling, Patrick G. Gavin, Casey M. Wright, Timothy D. Spector, Matthew A. Brown and Emma E. Hamilton-Williams

Figure S1

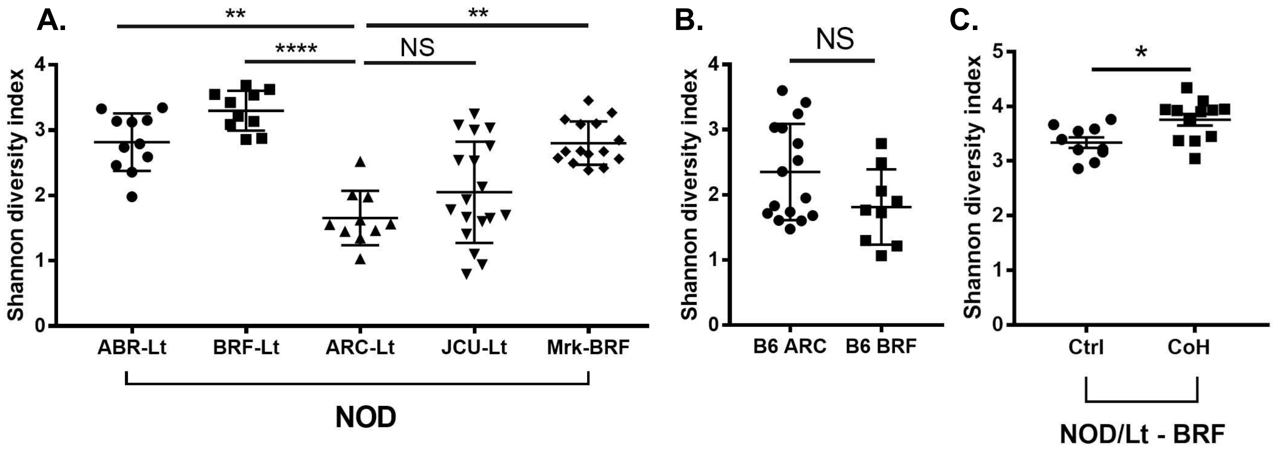

Figure S1. Microbiota alpha diversity differs between animal facilities but not consistently between genotypes. Shannon diversity index is shown for (A); NOD/Lt and NOD/Mrk mice from either ABR, ARC, JCU or BRF facilities (B); B6 from ARC or BRF. (C) NOD/Lt control mice and NOD/Lt co-housed with B6. (D); NOD, *Idd3,* I5, *Idd*3/5. (E); NOD and NOD.H-2^b. (F); NOD treated with either IL-2c or PBS. Mean and SD are shown.

Figure S2

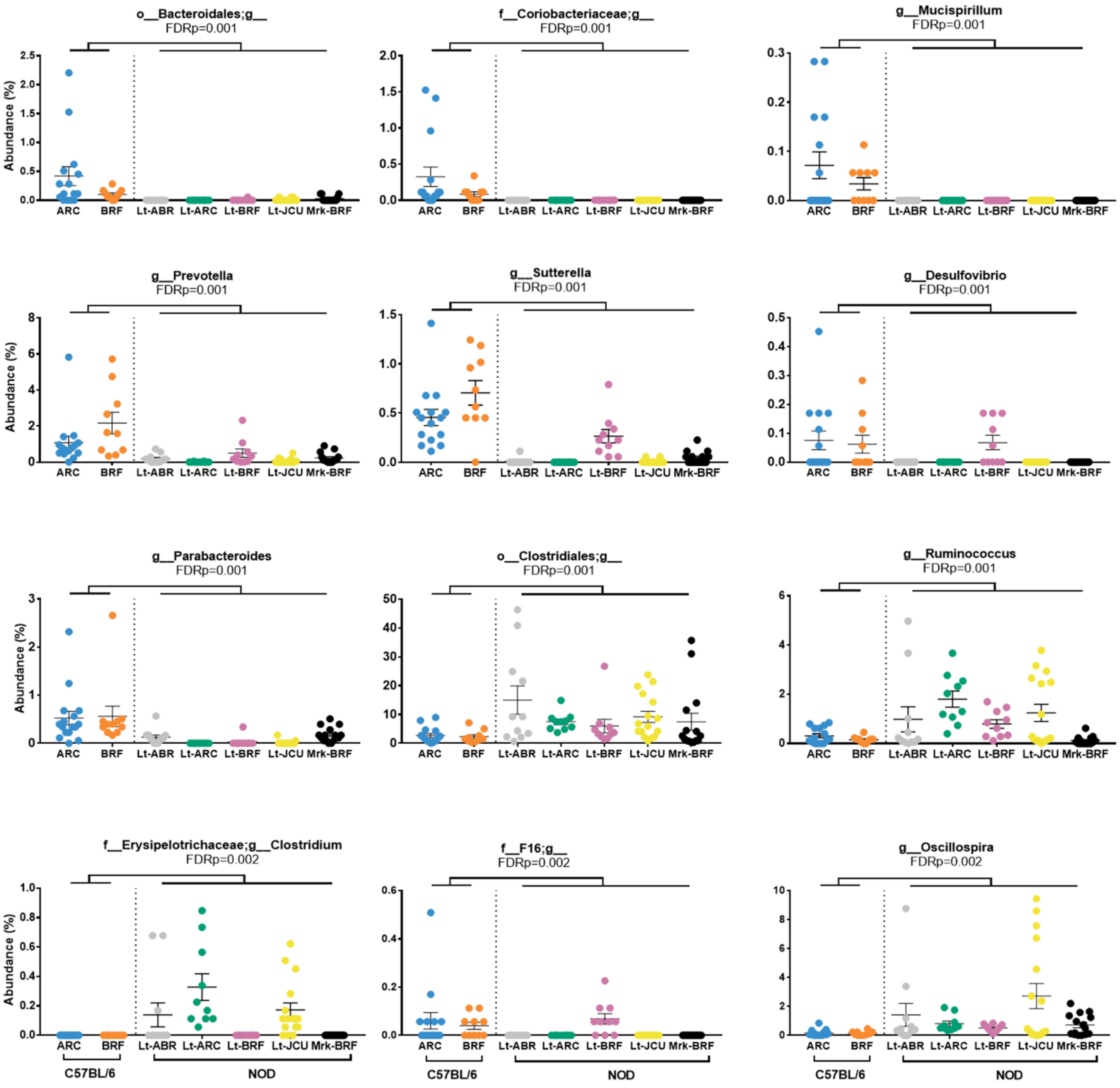

Figure S2. Significant differences in microbial taxa observed between NOD and B6 strains. Mean and SEM are shown for each group along with FDR adjusted p values.

Figure S3

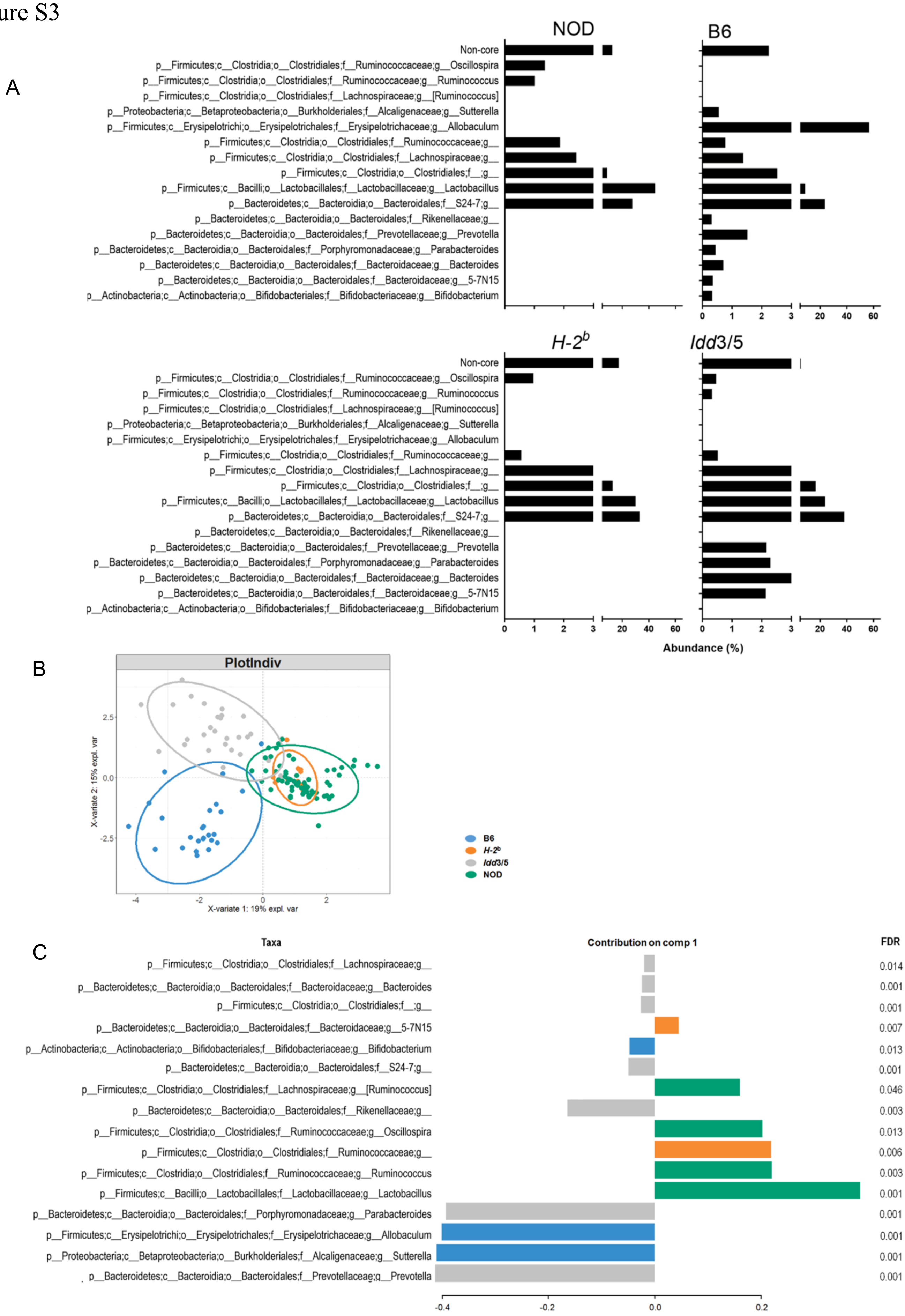

Figure S3. A core microbiome identified in NOD mice differs to B6 and *Idd*3/5 strains. A: The mean abundance of genera present in ≥80% of mice in each strain. B: sPLS-DA of the core microbiome genera. C: Component 1 contribution loadings from B. FDR indicates the adjusted P value of each genera between strains following permANOVA testing.

Figure S4

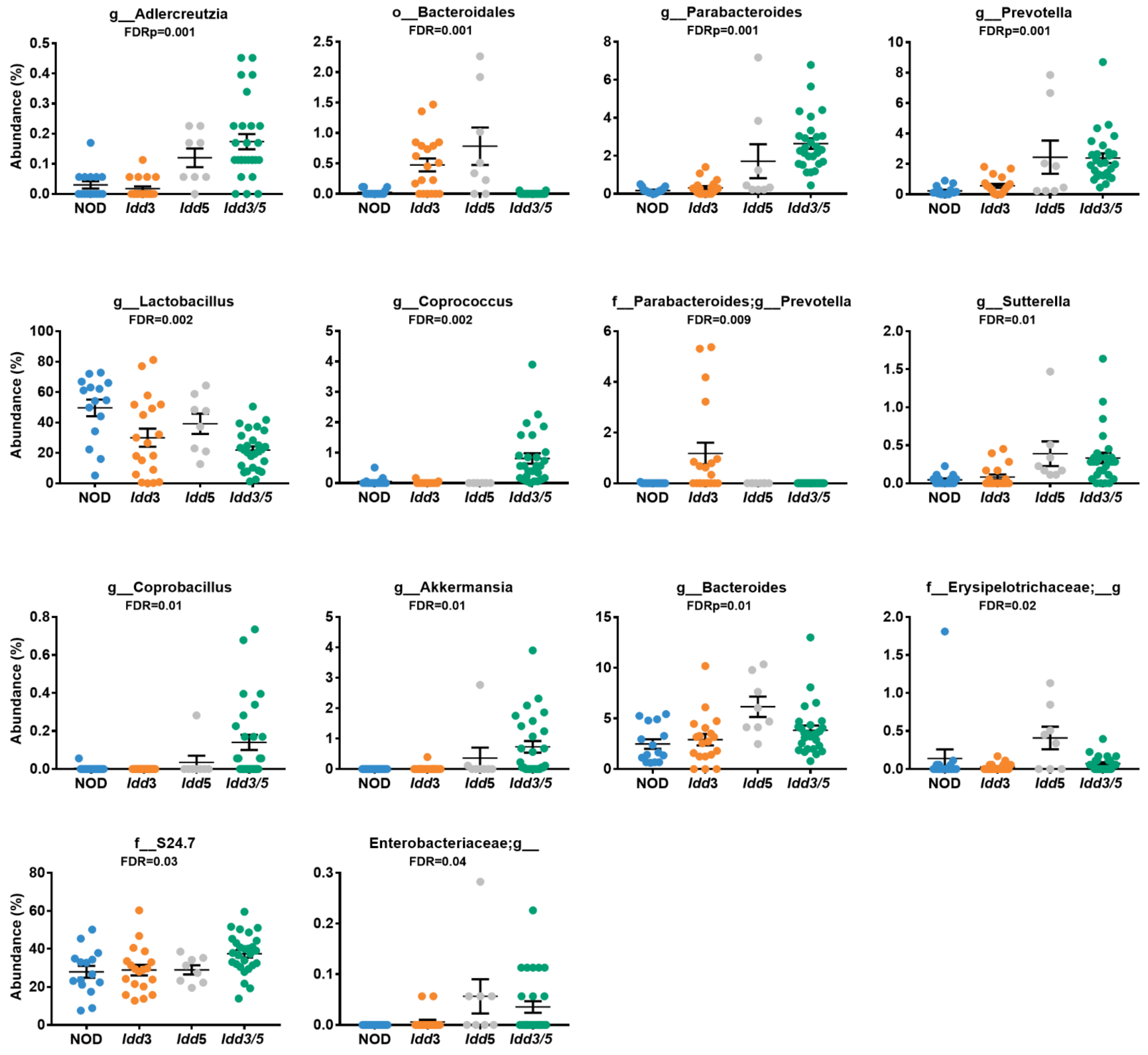

Figure S4. Significant differences in microbial taxa observed between NOD and *Idd*3/5 strains. Mean and SEM are shown.

Figure S5

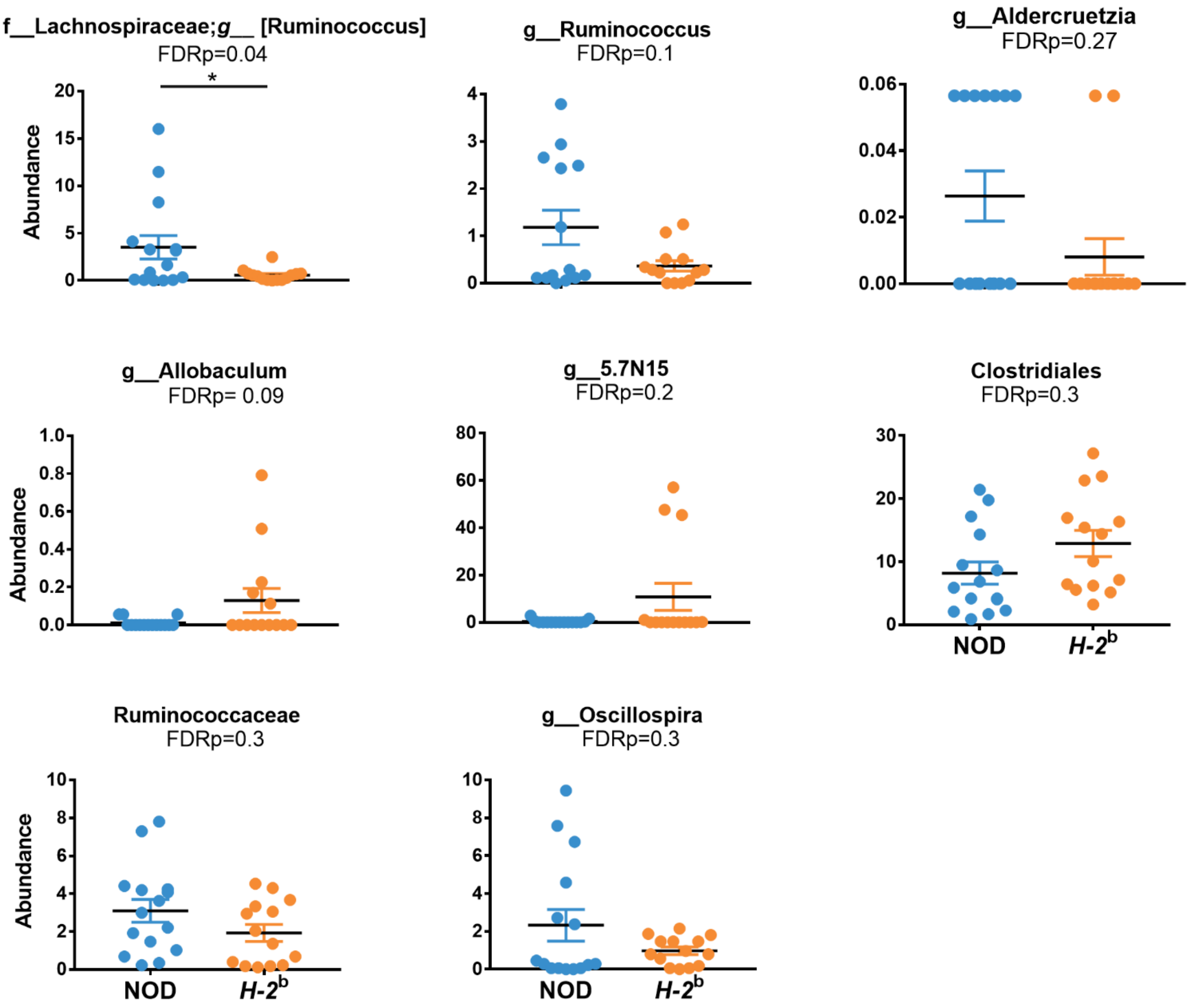

Figure S5. Microbial taxa contributing to variance between NOD.H-2^{b} and NOD mice.
Individual taxa contributing to component 1 loadings for the sPLS-DA analysis shown in Figure 2E. Mean and SEM are shown. FDR adjusted p values are shown.

Figure S6

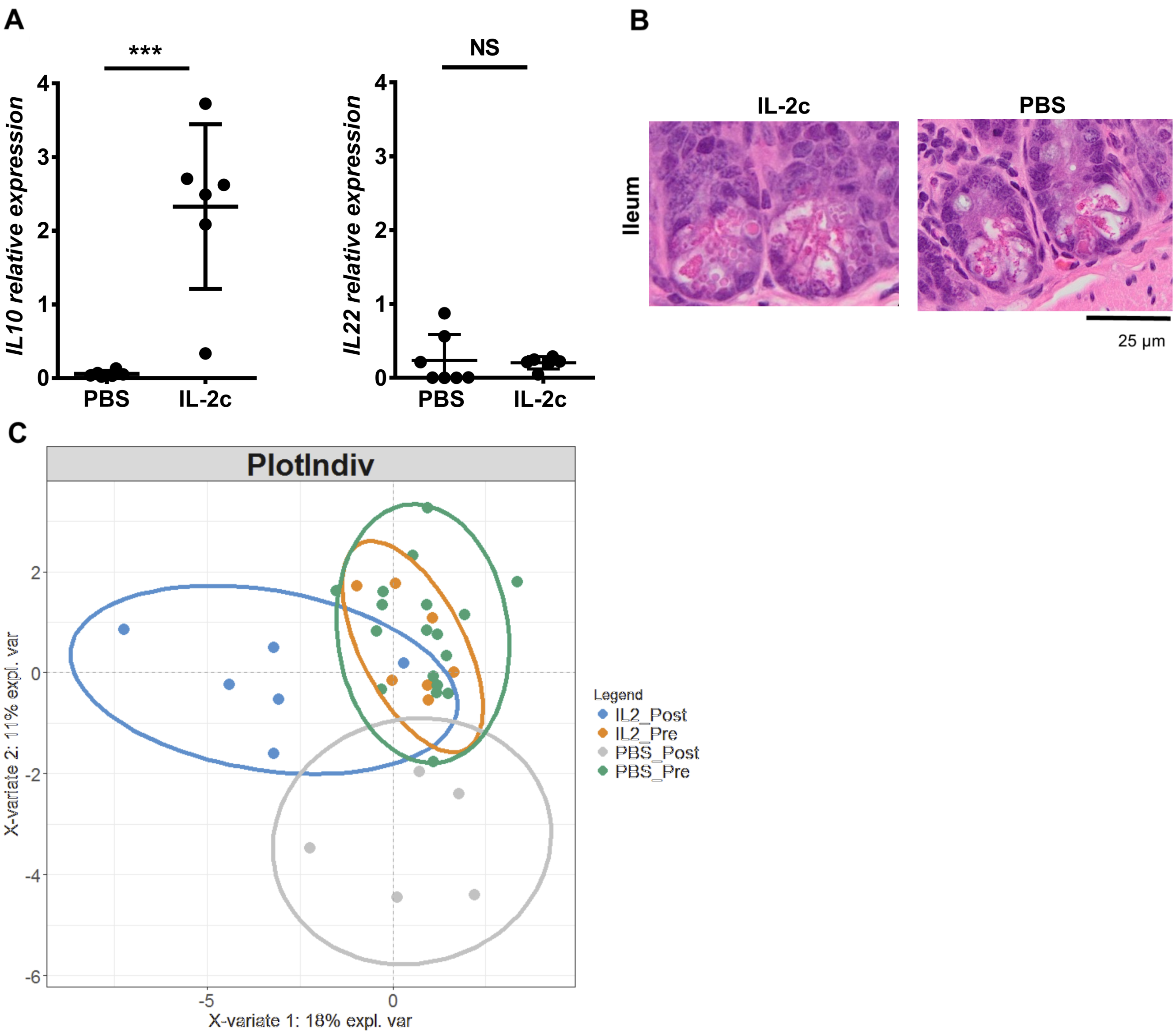

Figure S6. IL-2c treatment increases intestinal IL-10 production and alters the microbiota of NOD mice. NOD mice were treated with PBS or IL-2c weekly for 6 weeks. (A) *IL-10* and *IL-22* gene expression relative to *Hprt* in the ileum. (B) Representative images of H&E stained sections of ileum crypt termini showing Paneth cell morphology. (C) sPLS-DA multivariate analysis of fecal samples collected before treatment at 4-weeks of age (PBS_Pre and IL2_Pre) and after treatment at 12 weeks of age (PBS_Post and IL2_Post).
